# Regional Differences in Early BP Management After Acute Ischemic Stroke in the ENCHANTED International Randomized Controlled Trials

**DOI:** 10.3389/fneur.2021.687862

**Published:** 2021-08-27

**Authors:** Chen Chen, Lili Song, Jie Yang, Richard Lindley, Thompson Robinson, Hisatomi Arima, John Chalmers, Craig S. Anderson, Xia Wang

**Affiliations:** ^1^The George Institute for Global Health, Faculty of Medicine, University of New South Wales, Newtown, NSW, Australia; ^2^The George Institute China at Peking University Health Science Centre, Beijing, China; ^3^Department of Neurology, Shanghai East Hospital, School of Medicine, Tongji University, Shanghai, China; ^4^Department of Neurology, the First Affiliated Hospital of Chengdu Medical College, Chengdu, China; ^5^Sydney Medical School, University of Sydney, Sydney, NSW, Australia; ^6^Department of Cardiovascular Sciences and NIHR Biomedical Research Centre, University of Leicester, Leicester, United Kingdom; ^7^Department of Preventive Medicine and Public Health, Fukuoka University, Fukuoka, Japan; ^8^Department of Neurology, Royal Prince Alfred Hospital, Sydney, NSW, Australia

**Keywords:** stroke, thrombolysis, blood pressure, regional difference, clinical trial

## Abstract

**Background and Aims:** Epidemiological studies show significant variations in hypertension management within and between countries. The level of regional variation in early blood pressure (BP) management after acute stroke is uncertain.

**Methods:** Data are from the Enhanced Control of Hypertension and Thrombolysis Stroke Study (ENCHANTED), a partial-factorial, international randomized controlled trial of thrombolysis-eligible acute ischemic stroke (AIS) patients with elevated systolic BP (SBP >150 mmHg) assigned to intensive (target SBP 130-140 mmHg) vs. guideline-recommended (SBP <180 mmHg) treatment; BP management was compared among four regions: Western countries (Italy/United Kingdom/Spain/Australia), China (mainland), other Asia (Hong Kong/Taiwan/Singapore/Thailand/Vietnam/India), and South America (Chile/Brazil/Colombia).

**Results:** These analyses included 2,196 AIS [38% women, mean age 67 (12) years] patients. Commonly used intravenous BP-lowering agents were labetalol, nitroglycerin, and topical nitrates in Western countries; urapidil and sodium nitroprusside in China; nicardipine in other Asian countries; and sodium nitroprusside and labetalol in South America. Chinese patients were less likely to receive BP-lowering treatment in the first 24 h and be treated with multiple agents although they had smaller magnitude of SBP reduction and lower SBP variability.

**Conclusion:** Regional variations in early BP management in acute stroke translated into differences in early BP control parameters.

## Introduction

Elevated blood pressure (BP) is one of the leading modifiable risk factors for stroke, including both intracerebral hemorrhage (ICH) and acute ischemic stroke (AIS) (1). In acute stroke, 75% of patients have high BP, and 50% of those have a prior history of hypertension (2, 3). Large-scale epidemiological studies demonstrate significant variations in hypertension management at the country level and that within countries (4, 5). However, evidence on the variation in early BP management after acute stroke is scarce.

In the context of thrombolysis and BP management in AIS, the Enhanced Control of Hypertension and Thrombolysis in Stroke Study (ENCHANTED) (6, 7) enrolled patients at 110 hospitals in 14 countries. Most Asian participants were from middle income countries. This trial provides an ideal population to understand the regional differences in early BP management after AIS world widely.

## Materials and Methods

### Study Design

ENCHANTED (6, 7) was an international, 2 × 2 partial-factorial, multicenter, prospective, randomized, open-label, blinded-endpoint (PROBE) trial. All ENCHANTED participants had a clinical diagnosis of AIS that was confirmed by brain imaging, were aged ≥18 years, and fulfilled local criteria for thrombolysis treatment. In the BP arm, a total of 2,196 participants with systolic BP (SBP) >150 mmHg and time from onset within 6 h were randomly assigned to intensive (target SBP 130-140 mmHg) or guideline-recommended (SBP <180 mmHg) BP management between March 3, 2012, to April 30, 2018. In addition, the treating clinician had uncertainty over the benefits and risks of the intensity of BP control during and for up to 72 h (or hospital discharge or death if this occurred earlier) after thrombolytic treatment. A management strategy of BP-lowering treatment protocol in the ENCHANTED trial was based on locally available intravenous (IV, bolus and infusion), oral and topical medications (7). All patients were managed in an acute stroke unit or alternative environment with appropriate staffing and monitoring and received best practice management according to local guidelines. The use of endovascular thrombectomy, which increased in clinical practice during the course of the trial, was permitted.

### Measurements

Countries were grouped into four regions: China (mainland), Western countries (United Kingdom, Italy, Norway, Australia), other Asia (South Korea, Hong Kong Area, Taiwan Area, Singapore, Thailand, Vietnam), and South America (Chile, Brazil, Colombia).

For each participant, summary measures of SBP control in the first 24 h were (i) “achieved SBP,” the mean of SBP measures within the 24 h (“achieved” for short); (ii) “variability of SBP,” the standard deviation (SD) of the measures within the 24 h (“variability” for short); (iii) “magnitude of absolute reduction of SBP,” the difference between randomization SBP and the lowest attained SBP within the 24 h (“magnitude” for short).

### Data Analysis

Generalized linear model (GLM) was used to investigate the difference in hemodynamic variables in the first 24 h between the four regions. The model was adjusted for age, sex, baseline SBP, baseline NIHSS score, comorbidities of prestroke disability, hypercholesterolemia, atrial fibrillation (AF), antithrombotic therapy, and randomized BP-lowering treatment. All statistical analyses were performed using SAS version 9·3 (SAS Institute, Cary, NC, USA).

## Results

A total of 2,196 ENCHANTED participants (38% female) of mean age 67 years (SD 12.2) were included in these analyses. Median time from symptom onset to randomization was 3.3 h (IQR 2.6 to 4.1). Key baseline characteristics are provided in Table 1. There were 302, 1,428, 192, and 274 patients from Western countries, China, other Asia, and South America, respectively. Patients from Asia were younger and less female. Chinese patients had more previous stroke and stroke due to significant intracranial atheroma but were less likely to have history of AF and hypercholesterolemia, have strokes due to cardio embolism, and take antithrombotic agents.

**Table 1:** Baseline characteristics.

	**Western countries**	**China**	**Other Asian**	**South America**	***P*** **-value**
Time from the onset of symptoms to randomization, h	3.1 (2.3–4.0)	3.4 (2.7–4.1)	3.3 (2.6–4.0)	3.3 (2.4–4.2)	0.025
Demography					
Sex, female	135/302 (44.7)	512/1,428 (35.9)	70/192 (36.5)	118/274 (43.1)	0.008
Age, years	74.2 (11.9)	65.4 (11.4)	64.5 (12.8)	68.4 (12.9)	<0.0001
≥80	110/302 (36.4)	134/1,428 (9.4)	21/192 (10.9)	54/274 (19.7)	<0.0001
Clinical features					
Systolic BP, mmHg	168.5 (8.2)	164.3 (9.0)	166.3 (10.6)	166.2 (9.1)	<0.0001
Diastolic BP, mmHg	85.6 (13.3)	92.1 (10.9)	90.9 (10.4)	90.6 (11.0)	<0.0001
Heart rate, beats per minute	79.4 (17.6)	78.4 (13.5)	85.0 (14.2)	80.0 (17.1)	<0.0001
NIHSS score^*^	7.0 (4.0–12.0)	7.0 (4.0–12.0)	9.0 (6.5–12.5)	7.0 (5.0–13.0)	<0.0001
GCS score^†^	15.0 (14.0–15.0)	15.0 (14.0–15.0)	15.0 (15.0–15.0)	15.0 (13.0–15.0)	0.0002
Medical History					
Hypertension	195/302 (64.6)	1,019/1,425 (71.5)	140/191 (73.3)	214/274 (78.1)	0.004
Currently treated hypertension	173/302 (57.3)	571/1,425 (40.1)	81/191 (42.4)	187/274 (68.2)	<0.0001
Previous stroke (ischemic, hemorrhagic, or uncertain)	45/302 (14.9)	299/1,428 (20.9)	23/192 (12.0)	47/274 (17.2)	0.004
Coronary artery disease	39/302 (12.9)	212/1,425 (14.9)	15/191 (7.9)	43/274 (15.7)	0.051
Other heart disease (valvular or other)	17/302 (5.6)	40/1,425 (2.8)	11/191 (5.8)	26/274 (9.5)	<0.0001
Atrial fibrillation confirmed on electrocardiogram	60/301 (19.9)	175/1,424 (12.3)	23/191 (12.0)	54/274 (19.7)	0.0002
Diabetes mellitus	59/302 (19.5)	318/1,425 (22.3)	37/191 (19.4)	82/274 (29.9)	0.011
Hypercholesterolemia	104/302 (34.4)	46/1,425 (3.2)	23/191 (12.0)	76/274 (27.7)	<0.0001
Current smoker	42/300 (14.0)	318/1,425 (22.3)	29/191 (15.2)	55/274 (20.1)	0.003
Estimated pre-morbid function (mRS)					
No symptoms (score 0)	213/302 (70.5)	1,284/1,424 (90.2)	178/191 (93.2)	202/274 (73.7)	<0.0001
Symptoms without any disability (score 1)	89/302 (29.5)	140/1,424 (9.8)	13/191 (6.8)	72/274 (26.3)	
Medication at time of admission					
Warfarin anticoagulation	10/302 (3.3)	10/1,425 (0.7)	2/191 (1.0)	7/274 (2.6)	0.001
Aspirin or other antiplatelet agent	107/302 (35.4)	162/1,425 (11.4)	34/191 (17.8)	83/274 (30.3)	<0.0001
Statin or other lipid lowering agent	50/302 (16.6)	169/1,425 (11.9)	19/191 (9.9)	63/274 (23.0)	<0.0001
Presumed stroke etiology^‡^					
Large artery disease due to significant extracranial atheroma	26/300 (8.7)	73/1,399 (5.2)	10/188 (5.3)	40/273 (14.7)	<0.0001
Large artery disease due to significant intracranial atheroma	29/300 (9.7)	714/1,399 (51.0)	42/188 (22.3)	18/273 (6.6)	<0.0001
Small vessel disease	57/300 (19.0)	434/1,399 (31.0)	99/188 (52.7)	33/273 (12.1)	<0.0001
Cardioembolic	78/300 (26.0)	111/1,399 (7.9)	18/188 (9.6)	82/273 (30.0)	<0.0001
Dissection	1/300 (0.3)	1/1,399 (0.1)	1/188 (0.5)	4/273 (1.5)	0.003
Other or uncertain etiology	98/300 (32.7)	52/1,399 (3.7)	18/188 (9.6)	88/273 (32.2)	<0.0001

*Data are n (%), mean (SD), or median (IQR). P-values are based on Chi-square, T-test, or Wilcoxon signed-rank test*.

*BP, blood pressure; CT, computerized tomography; GCS, Glasgow coma scale; MRI, magnetic resonance imaging; mRS, modified Rankin scale; NIHSS, National Institutes of Health Stroke Scale*.

* *Scores on the National Institutes of Health stroke scale (NIHSS) range from 0 to 42, with higher scores indicating more severe neurological deficit*.

† *Scores on the Glasgow coma scale (GCS) range from 15 (normal) to 3 (deep coma)*.

‡ *Diagnosis according to the clinician's interpretation of clinical features and results of investigations at the time of separation from hospital*.

**Figure 1:**
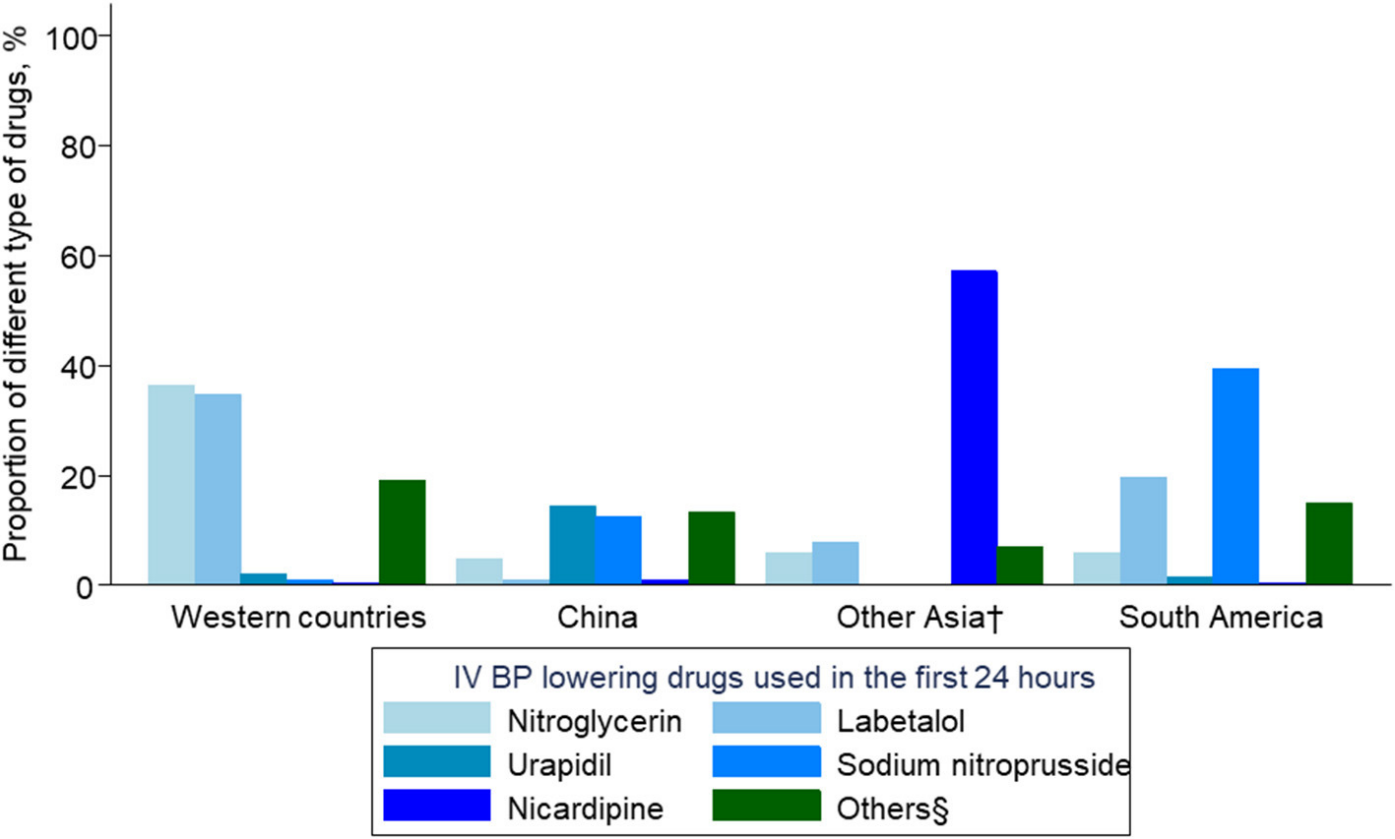
Intravenous blood pressure–lowering drugs used in the first 24 h. BP denotes blood pressure; IV intravenous. ^†^South Korea/Hong Kong area/Taiwan area/Singapore/Thailand/Vietnam. §metoprolol, atenolol, clevidipine, nimodipine, nifedipine, urapidil, isodinitrate, frusemide, prazosin, hydralazine, clonidine, and enalapril.

Patients from China, compared with other regions, were significantly less likely to receive BP-lowering treatment in both first 24 h and 2–7 days. In the first 24 h, the most popular IV agents in China were urapidil (14.5%) and sodium nitroprusside (12.4%). More than a third of the patients from Western countries (36.4%) were treated by IV nitroglycerin, followed by labetalol (34.8%). More than a half of the patients in other Asian countries received nicardipine (57.1%). In South America, most patients received sodium nitroprusside (39.4%) and labetalol (19.7%). For oral antihypertensive therapies, patients were more likely to take oral angiotensin converting enzyme (ACE) inhibitors/angiotensin-receptor blockers (ARBs) and calcium channel blockers in all four regions. The same pattern was also observed over 2–7 days (Figure 1 and Table 2).

**Table 2:** BP-lowering treatment by region in ENCHANTED.

	**Western countries**	**China**	**Other asian**	**South America**	***P*** **-value**
BP lowering in the first 24 h after randomization	241/302 (79.8)	867/1,412 (61.4)	149/191 (78.0)	203/274 (74.1)	<0.0001
Intravenous agent used, n(%)	176/302 (58.3)	661/1,412 (46.8)	133/191 (69.6)	190/274 (69.3)	<0.0001
Bolus on day 1	120/302 (39.7)	243/1,411 (17.2)	38/191 (19.9)	72/272 (26.5)	<0.0001
Infusion on day 1	102/302 (33.8)	430/1,411 (30.5)	124/191 (64.9)	142/273 (52.0)	<0.0001
Number of iv agents, n(%)					
0	126/302 (41.7)	772/141 2 (54.7)	64/191 (33.5)	87/274 (31.8)	<0.0001
1	115/302 (38.1)	451/1,412 (31.9)	109/191 (57.1)	147/274 (53.6)	<0.0001
2	53/302 (17.5)	138/1,412 (9.8)	16/191 (8.4)	34/274 (12.4)	<0.0001
>3	8/302 (2.6)	51/1,412 (3.6)	2/191 (1.0)	6/274 (2.2)	<0.0001
IV labetalol	105/302 (34.8)	11/1,412 (0.8)	15/191 (7.9)	54/274 (19.7)	<0.0001
IV nicardipine	1/302 (0.3)	14/1,412 (1.0)	109/191 (57.1)	1/274 (0.4)	<0.0001
IV urapidil	6/302 (2.0)	205/1,412 (14.5)	-	4/274 (1.5)	<0.0001
IV sodium nitroprusside	3/302 (1.0)	175/1,412 (12.4)	-	108/274 (39.4)	<0.0001
IV nitroglycerin	110/302 (36.4)	66/1,412 (4.7)	11/191 (5.8)	16/274 (5.8)	<0.0001
Other IV drug(s)	57/302 (18.9)	189/1,412 (13.4)	13/191 (6.8)	41/274 (15.0)	0.002
Oral agents used, n(%)					
0	127/127 (42.1)	920/920 (65.2)	126/126 (66.0)	214/214 (78.1)	<0.0001
1	79/206 (26.2)	345/1,265 (24.4)	32/158 (16.8)	34/248 (12.4)	<0.0001
2	54/260 (17.9)	120/1,385 (8.5)	30/188 (15.7)	14/262 (5.1)	<0.0001
*>3*	42/302 (13.9)	27/1,412 (1.9)	3/191 (1.6)	12/274 (4.4)	<0.0001
Oral ACEI/ARB	93/302 (30.8)	199/1,412 (14.1)	48/191 (25.1)	27/274 (9.9)	<0.0001
Oral diuretic	37/302 (12.3)	56/1,412 (4.0)	11/191 (5.8)	14/274 (5.1)	<0.0001
Oral beta blocker	49/302 (16.2)	87/1,412 (6.2)	8/191 (4.2)	14/274 (5.1)	<0.0001
Oral calcium channel blocker	69/302 (22.8)	294/1,412 (20.8)	30/191 (15.7)	29/274 (10.6)	0.0002
Other oral sympathetic antagonist	8/302 (2.6)	5/1,412 (0.4)	-	2/274 (0.7)	0.0001
Other oral medication(s)	70/302 (23.2)	25/1,412 (1.8)	5/191 (2.6)	15/274 (5.5)	<0.0001
BP lowering treatment in days 2–7					
Any BP lowering treatment, n(%)	247/299 (82.6)	857/1,394 (61.5)	151/188 (80.3)	206/273 (75.5)	<0.0001
Any intravenous BP lowering treatment, n(%)	82/299 (27.4)	492/1,394 (35.3)	71/188 (37.8)	115/273 (42.1)	0.0027
Bolus days 2–7	44/299 (14.7)	165/1,394 (11.8)	16/188 (8.5)	38/273 (13.9)	0.1662
Infusion days 2–7	51/299 (17.1)	323/1,393 (23.2)	61/188 (32.4)	85/273 (31.1)	<0.0001
Number of iv agents, n(%)					
0	219/219 (73.2)	920/920 (66.0)	121/121 (64.4)	163/163 (59.7)	0.0001
1	56/275 (18.7)	298/1,218 (21.4)	58/179 (30.9)	78/241 (28.6)	0.0001
2	19/294 (6.4)	132/1,350 (9.5)	6/185 (3.2)	19/260 (7.0)	0.0001
>3	5/299 (1.7)	44/1,394 (3.2)	3/188 (1.6)	13/273 (4.8)	0.0001
IV labetalol	32/299 (10.7)	5/1,394 (0.4)	5/188 (2.7)	26/273 (9.5)	<0.0001
IV nicardipine	-	7/1,394 (0.5)	55/188 (29.3)	-	<0.0001
IV sodium nitroprusside	-	88/1,394 (6.3)	1/188 (0.5)	63/273 (23.1)	<0.0001
IV nitroglycerin	58/299 (19.4)	64/1,394 (4.6)	6/188 (3.2)	14/273 (5.1)	<0.0001
Other IV drug(s)	35/299 (11.7)	252/1,394 (18.1)	10/188 (5.3)	40/273 (14.7)	<0.0001
Oral agents used, n(%)					
0	62/299 (20.7)	716/1,394 (51.4)	54/188 (28.7)	92/273 (33.7)	<0.0001
1	71/299 (23.7)	350/1,394 (25.1)	62/188 (33.0)	62/273 (22.7)	<0.0001
2	91/299 (30.4)	202/1,394 (14.5)	57/188 (30.3)	48/273 (17.6)	<0.0001
>3	75/299 (25.1)	126/1,394 (9.0)	15/188 (8.0)	71/273 (26.0)	<0.0001
Oral ACEI/ARB	145/299 (48.5)	350/1,394 (25.1)	94/188 (50.0)	135/273 (49.5)	<0.0001
Oral diuretic	60/299 (20.1)	158/1,394 (11.3)	25/188 (13.3)	71/273 (26.0)	<0.0001
Oral beta blocker	85/299 (28.4)	169/1,394 (12.1)	22/188 (11.7)	70/273 (25.6)	<0.0001
Oral calcium channel blocker	102/299 (34.1)	431/1,393 (30.9)	70/188 (37.2)	78/273 (28.6)	0.1667
Other oral sympathetic antagonist	9/299 (3.0)	14/1,394 (1.0)	-	15/273 (5.5)	<0.0001
Other oral medication(s)	104/299 (34.8)	45/1,394 (3.2)	12/188 (6.4)	32/273 (11.7)	<0.0001
BP lowering treatment at day 90					
Any BP lowering treatment at day 90	214/266 (80.5)	872/1,316 (66.3)	134/169 (79.3)	209/235 (88.9)	<0.0001

*Data are n (%). P-values are based on Chi-square test*.

*BP, blood pressure; IV, intravenous; ACEI, angiotensin converting enzyme inhibitors; ARB, angiotensin-receptor blockers*.

**Figure 2:**
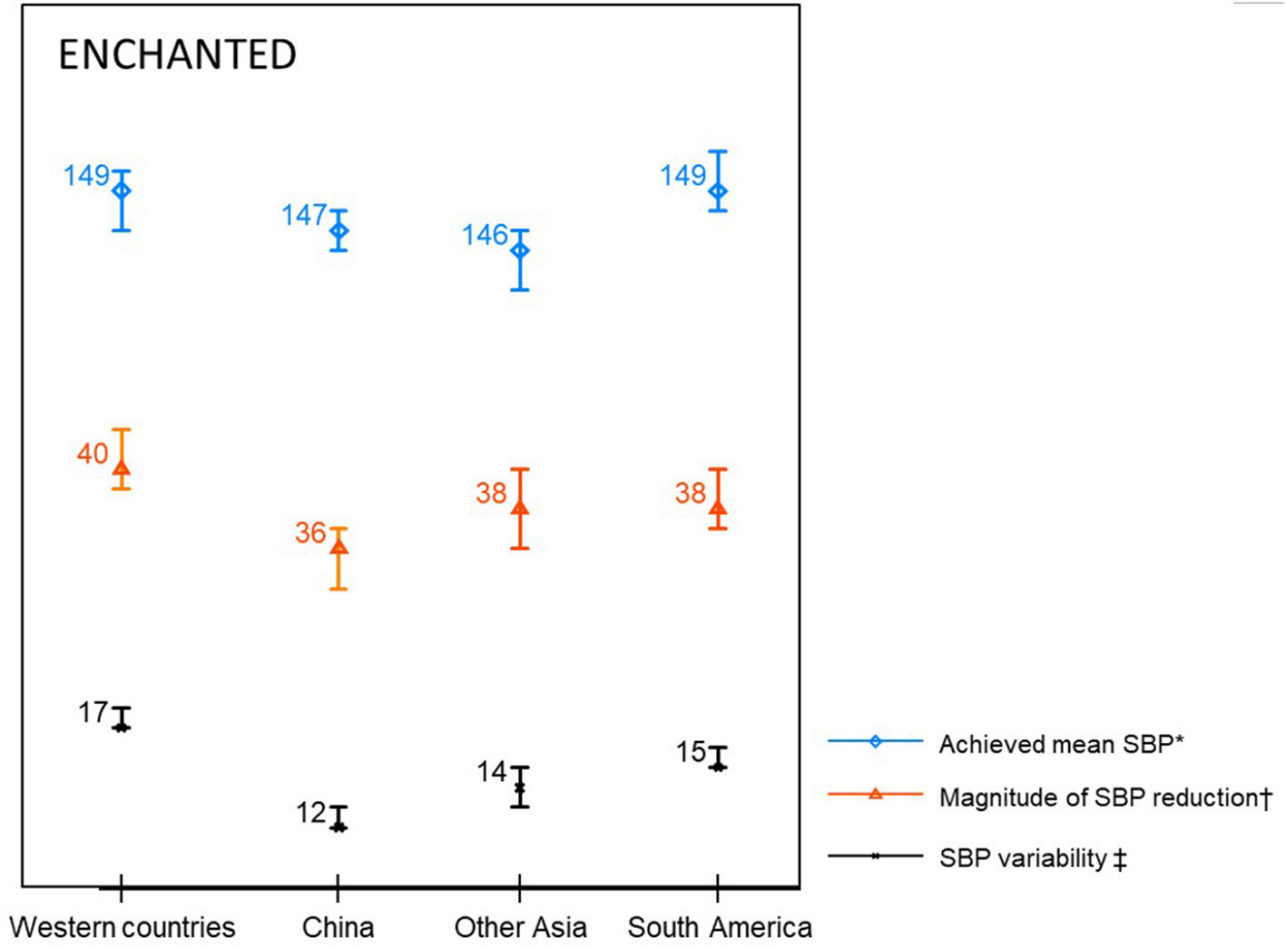
Blood pressure parameters in the first 24 h. SBP denotes systolic blood pressure. *Mean SBP in the first 24 h. ^†^SBP at randomisation minus minimum SBP within 24 h. ‡Standard deviation of SBP in the first 24 h.

Compared with other regions, patients from China had the least magnitude of SBP reduction and SBP variability throughout the first 7 days, and patients from Western countries had highest achieved mean SBP, magnitude of SBP reduction, and SBP variability over the first 24 h (Figure 2 and Table 3). Similar results were observed in both groups divided by randomization treatment (Supplementary Figure 1).

**Table 3:** BP control by region in ENCHANTED.

		**Western countries**	**China**	**Other asian**	**South America**	***P*** **-value**
Achieved mean SBP						
	Within 1 h	158 (157–160)	152 (150–153)	153 (151–155)	154 (152–156)	<0.0001
	Within 6 h	155 (154–157)	150 (149–151)	150 (148–152)	152 (151–154)	<0.0001
	Within 12 h	152 (151–153)	149 (147–150)	148 (146–150)	151 (149–152)	<0.0001
	Within 24 h	149 (147–150)	147 (146–148)	146 (144–147)	149 (148–151)	0.0005
	1–24 h	139 (138–141)	142 (141–144)	138 (136–140)	145 (143–146)	<0.0001
	2–7 days	138 (137–140)	141 (139–142)	136 (134–138)	140 (139–142)	<0.0001
Magnitude of SBP reduction						
	Within 1 h	23 (21–25)	22 (20–25)	24 (22–26)	18 (16–20)	<0.0001
	Within 6 h	29 (28–31)	29 (28–31)	31 (29–33)	31 (29–33)	0.082
	Within 12 h	36 (34–37)	33 (31–34)	35 (33–37)	35 (33–37)	0.0007
	Within 24 h	40 (39–42)	36 (34–37)	38 (36–40)	38 (37–40)	<0.0001
	1–24 h	39 (37–41)	34 (33–36)	37 (35–39)	36 (34–38)	<0.0001
	2–7days	37 (35–39)	37 (35–38)	42 (40–44)	40 (38–42)	<0.0001
Maximum SBP						
	Within 1 h	170 (168–172)	161 (160–163)	164 (161–166)	166 (164–168)	<0.0001
	Within 6 h	171 (169–173)	163 (161–164)	164 (162–167)	168 (166–170)	<0.0001
	Within 12 h	171 (170–173)	163 (162–165)	165 (163–167)	169 (167–171)	<0.0001
	Within 24 h	172 (171–174)	165 (163–166)	166 (163–168)	171 (169–173)	<0.0001
	1–24 h	163 (161–165)	157 (156–159)	155 (153–158)	164 (162–166)	<0.0001
	2–7 days	148 (147–150)	153 (151–154)	148 (146–150)	156 (154–157)	<0.0001
Minimum SBP						
	Within 1 h	147 (145–149)	142 (141–144)	143 (141–145)	141 (139–143)	<0.0001
	Within 6 h	136 (134–138)	136 (135–138)	134 (132–136)	134 (132–136)	0.082
	Within 12 h	130 (128–131)	133 (131–134)	130 (128–132)	130 (128–132)	0.0007
	Within 24 h	125 (123–127)	129 (128–131)	127 (125–129)	127 (125–129)	<0.0001
	1–24 h	126 (124–128)	131 (129–132)	128 (126–130)	129 (127–131)	<0.0001
	2–7 days	128 (126–130)	129 (127–130)	123 (121–126)	125 (124–127)	<0.0001
SBP variability (standard deviation)						
	Within 1 h	9 (8–9)	10 (9–11)	11 (11–12)	11 (11–12)	<0.0001
	Within 6 h	15 (14–16)	11 (10–11)	13 (12–14)	14 (13–15)	<0.0001
	Within 12 h	17 (16–17)	12 (11–12)	14 (13–15)	15 (14–16)	<0.0001
	Within 24 h	17 (17–18)	12 (12–13)	14 (13–15)	15 (15–16)	<0.0001
	1–24 h	15 (14–16)	11 (10–11)	11 (10–12)	14 (13–15)	<0.0001
	2–7 days	10 (9–11)	9 (9–10)	11 (10–12)	12 (12–13)	<0.0001

*Data are mean (SD), or median (IQR). P-values are based on T-test, or Wilcoxon signed-rank test*.

*BP, blood pressure, SBP, systolic blood pressure*.

## Discussion

Our data adds evidence to regional differences in early BP management after AIS with patients from China being less likely to be treated with IV BP-lowering agents and less aggressively treated when compared with other regions. There are a number of possible explanations for this.

First, this may reflect ethnic and/or regional differences in hypertension management for both primary (8, 9) and secondary prevention (10) of stroke that have been reported from large-scale epidemiological studies. The PURE study (10) surveyed 7,519 participants with either a previous chronic heart disease event or stroke for use of cardiovascular event-preventing drugs (β-blockers, statins, antiplatelet therapy, or BP-lowering drugs) for secondary prevention. Two thirds of the variation in antihypertensive drug use could be explained by the economic status of the country, whereas only a third by individual factors (10). The Global Burden of Disease Study 2013 (8) and INTERSTROKE study (9) showed that the population-attributable risk of stroke due to hypertension was higher in Asian populations than in those of other ethnicities or regions. There are uncertainties in worldwide AIS clinical guidelines for blood pressure control, especially within 48 h after onset (11, 12).

Second, compared with other regions, there is a large number of patients with intracranial artery stenosis in China. This may influence Chinese clinicians' decision-making with concerns related to high risk of early neurological deficit with antihypertensive treatment in the AIS period. Accordingly, patients from China had a smaller magnitude of SBP reduction and SBP variability over the first 24 h. This may relate to less use of BP-lowering treatment and less aggressive management with more than one type of IV agent. Overall, Chinese doctors are more conservative for BP-lowering treatment in acute stroke.

Third, Chinese patients were ~10 years younger than the Western patients and with fewer comorbidities, which may partly explain less need for BP-lowering therapy though these aspects were corrected in the multivariable analysis.

Finally, this may reflect regional differences in the use of IV BP-lowering agents. The lack of significant regional difference in oral BP-lowering drug use is likely to reflect drug class—thiazide diuretics, ACE inhibitors, calcium antagonists, ARBs, and beta blockers—are equally effective with respect to the magnitude of BP reduction (approximately 10/5 mm Hg) when administered separately (Evidence Grade 1) (13). However, there is debate about the differential effect on reduction in stroke recurrence, which may be related to differences in BP variability measurements.

Key strengths of this study are the international recruitment of patients with a broad range of characteristics and use of a robust protocol with central randomization and objective outcome measures. Nonetheless, no information on the dose of BP-lowering agents was collected. This is a mainly descriptive study and prone to inadequate adjustment. In addition, intensive BP lowering was the main intervention in ENCHANTED, and this could affect antihypertensive treatment performance. The proportion of antihypertensive treatment may be overestimated. However, no specific drug was specified in the protocol, and the results can still show the regional differences in BP management.

In summary, regional variations appeared in early BP management, including receiving BP-lowering treatment, the intensity of the treatment, and BP-lowering agents used in AIS, which translated into the differences in hemodynamic parameters. Furthermore, regional differences need to be considered when conducting BP management trials and formulating blood pressure management guidelines.

## Data Availability Statement

De-identified participant data used in these analyses can be shared by formal request to the corresponding author. Requests to access these datasets should be directed to Craig Anderson, canderson@georgeinstitute.org.au.

## Ethics Statement

The studies involving human participants were reviewed and approved by Australia Royal Prince Alfred Hospital HREC. The patients/participants provided their written informed consent to participate in this study.

## Author Contributions

LS, CA, and XW contributed to study design, organization, statistical review, and critique of the report, and XW undertook analyses. CC drafted the manuscript. All authors contributed to the concept and rationale for the study, made critical revisions, approved the final article, and take responsibility for its content and integrity.

## Author Disclaimer

The views expressed in this article are those of the author(s) and not necessarily those of the NIHR or Department of Health and Social Care.

## Conflict of Interest

LS, HA, and CA report speaking fees from Takeda China. TR is a National Institute for Health Research (NIHR) Senior Investigator; JC reports grants from Servier and NHMRC. CA holds a Senior Investigator Fellowship and grants from NHMRC and Takeda China. XW is supported by National Heart Foundation postdoctoral fellowship (102117); New South Wales Health commission investigator development grant; and NHMRC investigator grant (APP1195237). The remaining authors declare that the research was conducted in the absence of any commercial or financial relationships that could be construed as a potential conflict of interest.

## Publisher's Note

All claims expressed in this article are solely those of the authors and do not necessarily represent those of their affiliated organizations, or those of the publisher, the editors and the reviewers. Any product that may be evaluated in this article, or claim that may be made by its manufacturer, is not guaranteed or endorsed by the publisher.
