# Supplementary material for: Regional Differences in Early BP Management After Acute Ischemic Stroke in the ENCHANTED International Randomized Controlled Trials
Source: Front Neurol. 2021 Aug 27;12:687862. doi: 10.3389/fneur.2021.687862 (PMC8432933; doi:10.3389/fneur.2021.687862)
Supplement: Supplementary file 1 [file Data_Sheet_1.docx]

**Supplementary Figures**

**Regional differences in early BP management after acute ischemic stroke in the ENCHANTED international randomized controlled trials**

Chen Chen^1,2,3^, Lili Song,^1,2^ ^†^* Jie Yang,^4^ Richard Lindley,^1,5^ Thompson Robinson,^6^ Hisatomi Arima PhD,^7^ John Chalmers, ^1^ Craig S Anderson, ^1,2,8^ ^†^* Xia Wang.^1^ for the ENCHANTED Investigators

^1^The George Institute for Global Health, Faculty of Medicine, University of New South Wales, NSW, Australia

^2^The George Institute China at Peking University Health Science Centre, Beijing, PR China

^3^Department of Neurology, Shanghai East Hospital, School of Medicine, Tongji University, Shanghai, PR China

^4^Department of Neurology, the First Affiliated Hospital of Chengdu Medical College, Chengdu, China

^5^University of Sydney, Sydney, Australia

^6^Department of Cardiovascular Sciences and NIHR Biomedical Research Centre, University of Leicester, Leicester, UK

^7^Department of Preventive Medicine & Public Health, Fukuoka University Japan

^8^Department of Neurology, Royal Prince Alfred Hospital, Sydney, Australia

**Supplementary Figure 1** Blood pressure parameters in the first 24 hours by randomization group


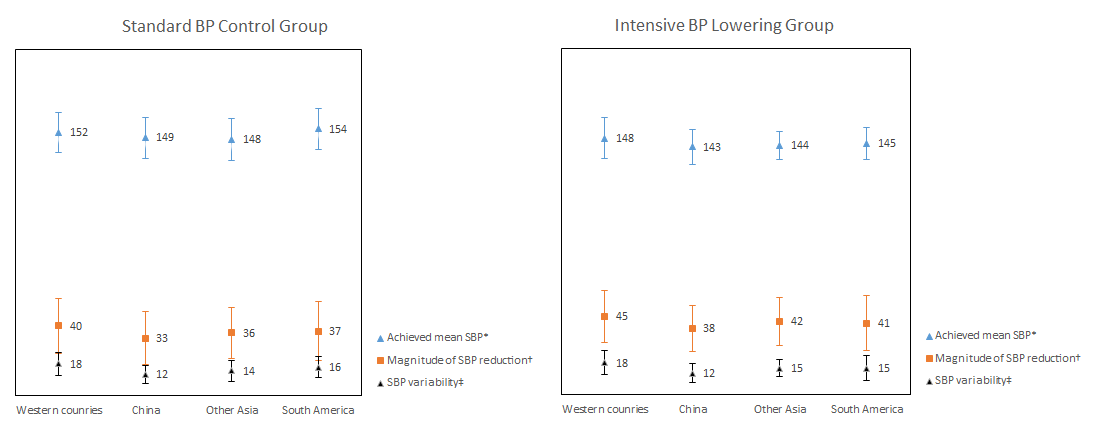


SBP denotes systolic blood pressure

*Mean SBP in the first 24 h

†SBP at randomisation minus minimum SBP within 24 h

‡Standard deviation of SBP in the first 24 h
